# An integrated network of Arabidopsis growth regulators and its use for gene prioritization

**DOI:** 10.1038/srep17617

**Published:** 2015-12-01

**Authors:** Ehsan Sabaghian, Zuzanna Drebert, Dirk Inzé, Yvan Saeys

**Affiliations:** 1 Department of Plant Systems Biology, VIB, 9052 Gent, Belgium; 2 Department of Plant Biotechnology and Bioinformatics, Ghent University, 9052 Gent, Belgium; 3 Inflammation Research Center, VIB, 9052 Gent, Belgium; 4 Department of Respiratory Medicine, Ghent University Hospital, Gent, Belgium

## Abstract

Elucidating the molecular mechanisms that govern plant growth has been an important topic in plant research, and current advances in large-scale data generation call for computational tools that efficiently combine these different data sources to generate novel hypotheses. In this work, we present a novel, integrated network that combines multiple large-scale data sources to characterize growth regulatory genes in Arabidopsis, one of the main plant model organisms. The contributions of this work are twofold: first, we characterized a set of carefully selected growth regulators with respect to their connectivity patterns in the integrated network, and, subsequently, we explored to which extent these connectivity patterns can be used to suggest new growth regulators. Using a large-scale comparative study, we designed new supervised machine learning methods to prioritize growth regulators. Our results show that these methods significantly improve current state-of-the-art prioritization techniques, and are able to suggest meaningful new growth regulators. In addition, the integrated network is made available to the scientific community, providing a rich data source that will be useful for many biological processes, not necessarily restricted to plant growth.

Size control of multi-cellular organisms poses a longstanding biological question that has fascinated scientists from every time and generation. Currently, the mechanism behind size measurement and fixation during growth of an organ or organism is far from being resolved, essentially because of its complex, integrated nature of regulation at the cellular, tissue, organ and whole-organism level^1^,^2^,^3^. Due to the importance for food and renewable energy sources, dissecting the genetic networks underlying plant growth under both favorable as well as environmentally limiting conditions, is becoming a high priority of plant scientists worldwide^4^,^5^.

To model plant organ growth, we focus here on leaf growth in Arabidopsis. By using sunlight very efficiently as energy source to capture carbon dioxide and to build sugars, leaves have an indispensable role in providing ecosystems with energy and chemical building blocks. Leaves also have a pivotal role in crop productivity and, therefore, understanding how they grow and reach their final size is of high scientific interest. Arabidopsis leaf growth is a well-established experimental system to understand the regulatory networks governing organ size control, and during the last decade, numerous genes that regulate leaf size in Arabidopsis, as well as in other plants, have been identified^2^,^6^,^7^. At the same time, it has been recognized that growth regulatory networks are highly complex and involve many components that remain to be identified. Current advances in systems biology can significantly contribute to complete such networks^8^.

Within the field of systems biology, gene prioritization techniques refer to a class of computational methods that can be used to rank a set of genes with respect to a certain criterion of relevance^9^. Typically, such criteria concern the involvement in a particular biological process (such as leaf growth) or relatedness to a certain disease. Whereas many gene prioritization techniques exist in the medical field, usually attempting to find novel, disease-related genes, gene prioritization techniques in the plant sciences are still in their infancy. Computational strategies for gene prioritization in the medical field can be largely divided into two classes: filtering strategies and ranking strategies^10^. While filtering strategies reduce the set of candidate genes by applying filtering steps such as gene function or association status to obtain a small set of candidate genes, ranking strategies sort all candidate genes from most promising to least promising. The latter category allows for a finer analysis of the candidates, thereby avoiding the use of hard thresholds used in filtering strategies.

Ranking strategies can be further subdivided into text mining methods, similarity profiling, and network-based methods. Text mining methods typically start from a chosen set of keywords, subsequently retrieve the relevant literature, and extract from this the candidate genes that can be linked to the keywords. Statistical measures are then used to rank these candidate genes. A major limitation of this approach is the fact that only obvious genes will be found, and chances are low to find truly novel findings. Similarity profiling combines both knowledge bases and raw data, and uses these to measure the similarity of the candidate genes to a set of known genes. Subsequently, data fusion approaches are used to combine all similarity scores of all data sources into a global ranking. Finally, network-based methods use a network representation to combine all data sources, representing candidate genes as nodes and similarities based on different data sources as edges in the network. Graph-based algorithms are then used to rank all nodes in the network with regard to an initial subset of nodes (genes) known to be involved in the biological process or disease of interest^11^.

Currently, more than 33 gene prioritization tools are being used in the medical field^12^,^13^, whereas only two major prioritization tools for plants exist: AraNet^14^ and GeneMANIA^15^, both using a network-based approach for gene prioritization. AraNet^14^ is a probabilistic, integrated network of two main association data sets: comparative genomics data and proteomics data. AraNet first scores each network based on its ability to correctly reconstruct shared membership in Arabidopsis biological processes. Subsequently, all data is integrated into a single integrated network that contains 1,062,222 links among 19,647 genes. Each link is weighted by estimating the association between each pair of genes, defined as the likelihood of participating in the same process. The prioritization approach ranks genes by summing the scores of those links that are related to a set of query genes. Genes with high scores are strongly connected to the set of query genes and can be considered as the most likely new candidates.

GeneMANIA^16^ uses a combination of different data sets to find the genes that are most related to a set of query genes. The combined data set consists of genetic interactions, pathways, co-expression, co-localization and protein domain similarity. Two steps describe the whole procedure in GeneMANIA. In the first step, a variation of ridge regression is used to integrate multiple association networks. Subsequently, in the prediction step, a label propagation algorithm is used to rank genes in function of their relation to the query genes. GeneMANIA covers 10,244,303 edges between 24,815 Arabidopsis genes^15^.

Comparing both approaches, it is clear that GeneMANIA covers more Arabidopsis genes and also contains a richer network to find associations between genes. Another difference concerns the prioritization algorithm; whereas the prioritization in AraNet is based upon the principle of guilt-by-association, using only direct connections, GeneMANIA uses a more advanced strategy, taking into account global connectivity between genes and also further exploiting indirect connections in the network. The guilt-by-association approach used by AraNet has been shown to be problematic when dealing with genes having multiple functions, and is also an error-prone approach in a large-scale network context^17^. By looking at global connectivity, GeneMANIA determines a measure of similarity between each gene and the set of query genes based on the topology of the network. However, GeneMANIA does not explicitly look at specific, local topological patterns that could be useful to better characterize the query genes, and thus improve the prioritization.

In this work, we describe an in-depth analysis of novel network-based prioritization approaches for ranking Arabidopsis growth-regulating (*GR*) genes. At the data level, our work contributes two new network resources, based on state-of-the-art machine learning methods for transcriptional regulatory network inference on one hand, and event extraction from literature data on the other hand. These novel data sources were further combined with existing resources into a network representation. At the algorithmic level, a large variety of both existing and newly proposed connectivity patterns was subsequently extracted from the combined network, and machine learning methods were used to suggest new growth regulators (GRs). Using a large-scale comparison, these newly developed methods were able to outperform current network-based prioritization methods, and to suggest new, meaningful GRs.

The individual and combined networks, as well as all rankings, are publicly available at http://bioinformatics.psb.ugent.be/supplementary_data/ehsab/gene_prioritization/.

## Results and Discussion

### An integrated network of leaf growth regulators in Arabidopsis

We constructed an integrated network from seven different sub-networks, including both publicly available networks, as well as newly constructed networks that are made available to the community (Table 1). The AGRIS sub-network consists of known transcriptional regulatory links in *Arabidopsis thaliana*, and was obtained from the Arabidopsis Gene Regulatory Information Server (AGRIS)^18^. The MaMut genetic modification design sub-network contains information about differentially expressed genes when comparing wild-type plants to transgenic plants, and was obtained from the “genetic modification data set” of CORNET, a publicly available database of gene associations in plants^19^. The PPI sub-network was also obtained from CORNET, and consists of both predicted as well as experimentally identified protein-protein interactions (PPIs). The PCC and GENIE3 sub-networks were obtained from the curated “leaf” microarray compendium, also available in CORNET and which is a curated and filtered compendium, specifically grouping experiments done on Arabidopsis leaves. From this microarray compendium, we constructed a gene co-expression network, containing only the most correlated genes (Pearson Correlation Coefficient or PCC sub-network), as well as a transcriptional regulatory network (GENIE3 sub-network). The GENIE3 sub-network was constructed using a network inference tool (GENIE3)^20^, which achieved the best performance in the DREAM5 network inference challenge^21^. The GeneMANIA sub-network was also used, in itself consisting of a combination of different publicly available data sets, collected from a variety of databases^16^, and finally, a sub-network representing gene-gene associations predicted using text mining algorithms was extracted from the EVEX resource, which is built on top of the PubMed literature^22^. In this work, we only used gene-gene associations from EVEX that were annotated as belonging to Arabidopsis genes.

In this integrated network, genes were represented by nodes and biological relationships between genes by edges. To explore this integrated network, network topology properties were used. The resulting integrated network was a multiple-connected, undirected network without self-loops, consisting of 27,376 nodes (genes) and 24,361,406 edges. Table 1 depicts the global network properties of all individual sub-networks, as well as their integration. The largest sub-network in terms of edges was the network based on co-expression (PCC), while the GeneMANIA sub-network contained the highest number of nodes.

The GENIE3 and EVEX sub-networks are two novel networks introduced in this study, representing inferred networks of respectively predicted transcription factor-target relations from microarray data and relations extracted from PubMed texts using state-of-the-art text mining algorithms. While the GENIE3 sub-network covered the majority of all genes (21,503 nodes) and a substantial amount of edges, the EVEX sub-network constituted the smallest network, covering only about 10% of all Arabidopsis genes.

Overall, the integrated network was sparsely connected, showing a network density (ratio of the actual number of edges to the number of possible edges) of 0.065 and containing a limited overlap between the constituting sub-networks. 92% (22,309,895) of the edges were unique to one of the sub-networks, which means that only 8% (2,051,511) of the edges were part of two or more sub-networks. The diameter (longest geodesic distance between two nodes in the network) was 11 and the average shortest path between each two nodes was 2.13.

### Connectivity patterns of Arabidopsis leaf growth regulators

In a next step, we defined our biological process of interest as leaf growth in Arabidopsis. To this end, we defined a reference set of genes related to leaf growth consisting of two groups of genes. The first set of 57 genes (referred to as Intrinsic Yield Genes) are known to have a proven effect on leaf size when modified (available from http://www.yieldbooster.org/), the functions of which include regulation of cell size and/or cell number. The second set consisted of 98 putative growth-related genes selected from genome-wide transcript profiling of developing leaves, derived from an independent microarray experiment^23^ (see Methods). The two sets of *GR* genes showed 8 common genes, resulting in a total set of 147 *GR* genes (Supplementary Table S1). The remaining 27,229 genes out of the total set of 27,376 analyzed Arabidopsis genes were then further considered as the set of genes to be prioritized, in order to suggest potential novel GRs.

Comparing the Arabidopsis *GR* genes with the remaining genes of the network showed that their degree distribution was shifted toward higher numbers (Supplementary Fig. S1A), implying that *GR* genes were thus more connected to other genes than non-*GR* genes, and they were also more strongly connected to each other than to non-GR genes (Supplementary Fig. S1B). Also in terms of shared neighbors, Arabidopsis *GR* genes were more connected to each other than to other genes (Supplementary Fig. S1C).

**Figure 1:**
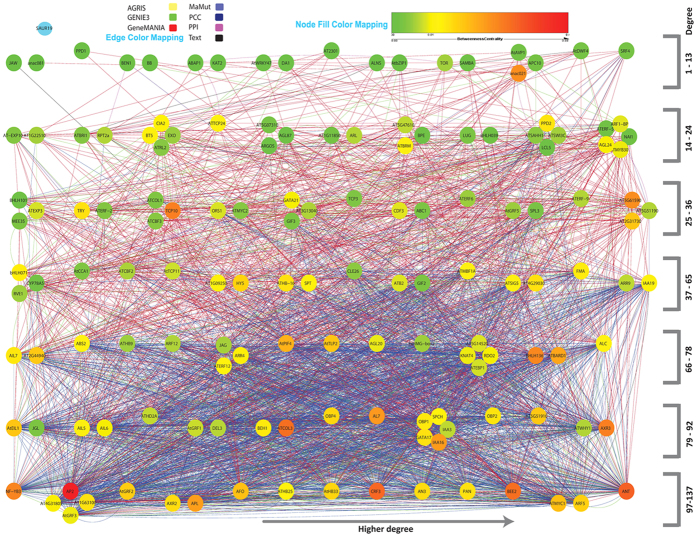
Local Network of *GR* genes. Graph structure of the *GR* genes based on the degree of interconnectivity, with nodes lower in the network having a higher degree. In the same layer, nodes are organized from left to right with increasing degree of interconnectivity. The color of the nodes shows the betweenness centrality (ability of nodes to keep the network connected).

Figure 1 shows a graphical representation of the interconnectivity in the local network connecting only the *GR* genes to each other. Genes are grouped into horizontal layers, with layers at the bottom having higher degrees of interconnectivity. Within each layer, genes situated toward the right have higher degrees of interconnectivity. Colored edges between genes show the different sub-network types, whereas node colors indicate the betweenness centrality of each gene, a measure of how important the gene is in connecting subparts of the network. In this local network, *ANT* was the most connected gene (138 edges), followed by *ARF5* and *MYC1* (both 117 edges). On the other hand, *SAUR19* was not connected to any other *GR* gene, and *JAW* and *ANAC081* had only one edge connecting them to other *GR* genes. In terms of centrality in the local network, *AP2* was the most important gene, whereas *JAW*, *ANAC081* and *PPD1* were the least central genes in the network. A special case was *ANAC021*, which – despite its low degree of connectivity (10 edges) – still had a large effect on the network, as shown by its relatively high betweenness value. The underlying reason for this is the fact that *ANAC021* plays a key role in connecting nodes mainly connected by the PCC sub-network to nodes mainly connected by the GeneMANIA sub-network. Also when looking at the edge and node betweenness values for the local network of *GR* genes (Supplementary Fig. S2), the link between *ANAC021* and *AP2* was of high importance, as well as the few links that connected low-degree nodes to the rest of the network (e.g. *JAW*, *PPD1*).

In the global network connecting all genes, *HD2A*, encoding a histone deacetylase, was interestingly the most connected growth regulator (8084 edges), while *JAW*, encoding *miR319a*, was again the least connected growth regulator with only three edges, coming from the text mining network. The importance of histone acetylation/deacetylation in regulating transcription and development is well documented (for a recent review, see Liu, *et al*.^24^). The median of the number of direct edges to *GR* genes was 38 for *GR* genes internally, and 7 for other genes. However, 5,078 of the non-growth regulators had at least 38 direct edges to the set of 147 *GR* genes, showing that there is a huge number of genes that is directly connected to *GR* genes. Therefore, only relying on the direct connection, the so-called “guilt-by-association” principle can be estimated to be insufficient to prioritize new *GR* genes.

### Network contribution

Looking at the contribution of each sub-network to both the local network, composed of *GR* genes only, as well as the extended network, considering *GR* genes and their first neighbors (Table 2), it could be observed that most of the edges were contributed by the PCC and the GeneMANIA sub-networks, which were also the largest networks. When taking only the local growth regulatory network into account, about half of the edges were contributed by the PCC sub-network, followed by the GeneMANIA (30%) and the GENIE3 (10%) sub-networks. To interpret the relative importance of each sub-network better, we normalized these numbers according to the total number of edges in each sub-network, in order to obtain relative contribution percentages (Table 2, column “Percent Individual”). In the local growth regulatory network, the PCC and GeneMANIA sub-networks only showed a relative contribution of 0.02% and 0.01%, respectively, whereas e.g. the EVEX and GENIE3 sub-networks obtained much higher relative contributions (0.57% and 0.13%, respectively). For the extended network that also takes into account first neighbors of *GR* genes, even 7.56% (EVEX) and 10.48% (GENIE3) of all edges contributed to the network, showing their efficiency.

### Network-based prioritization

#### Model-based prioritization

**Figure 2:**
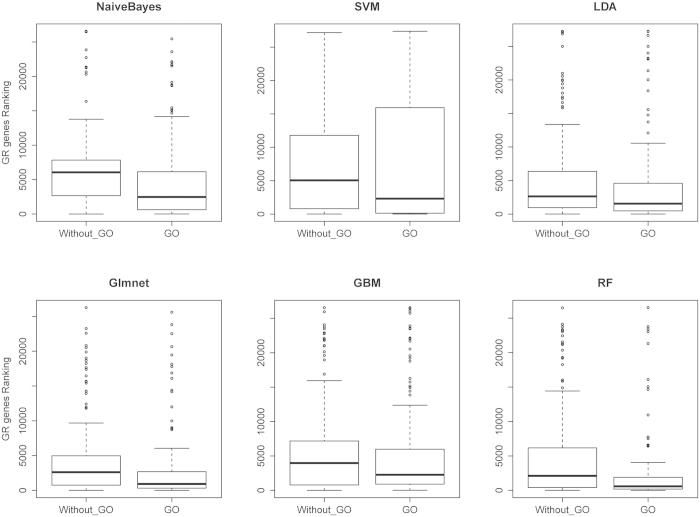
The Effect of GO Term Features on Classifier Performance. The effect of the inclusion of GO terms when using model-based approaches. By adding GO similarity scores as a new feature to the model-based approaches, all of them improved their ability in order to rank more *GR* genes on the top list. Each box plot shows the ranking of all 147 *GR* genes in the list of 27,290 genes. The approach that gives lower ranks to *GR* genes has a box plot shifted more towards zero on the y axis.

We trained a number of well-known machine learning methods, including Naïve Bayes (NB), Linear Discriminant Analysis (LDA), Support Vector Machines (SVM), Lasso and elastic-net regularized generalized linear models (Glmnet), Random Forest (RF), and Generalized Boosted Regression Models (GBM), to learn the mapping between network-based properties and involvement in growth regulation. Two classes of features were used: network-based features and Gene Ontology (GO)-derived features. Figure 2 displays the comparison of the results using a) only the network-based features (without_GO), and b) including also the GO-based features. Including the GO-based features within the model-based approaches clearly boosts their ability in predicting *GR* genes in a leave-one-out cross-validation (LOOCV) scheme (see Methods). For all methods, this resulted in a lower median rank and likewise, a lower first quartile, which is the most important part of the ranking if genes are to be evaluated in a top-down fashion (Table 3).

In terms of median ranking, the best results were obtained by the RF model (median rank of 589), which also had the lowest inter-quartile range (IQR) and ranks most genes within the first quartile. Interestingly, the best result for the first quartile (the top of the ranking) was obtained using the SVM method, which obtained a first quartile rank of 127 and was able to rank almost 30% of the *GR* genes within this first quartile. The two other methods that were able to rank a large number of *GR* genes within the first quartile were RF (20.3%) and Glmnet (13.3%). These results, especially the SVM approach, were encouraging and reliable enough to be used as automated prioritization techniques.

In addition to building separate models for gene prioritization, we also examined combinations of models, an approach referred to as ensemble models. Overall, no major improvements were noted by combining methods, and the best combination was only able to marginally improve the median ranking (RF combined with Glmnet, see Table 3). The implementation of the ensemble models and their results are available in the Supplemental Information (Supplementary Fig. S3).

To test the sensitivity of the results to the chosen cross-validation setup, we repeated the analysis using 10-fold cross-validation. In the latter setup, instead of leaving out each time only one gene as a test set, 10% of the *GR* genes were left out for testing, thus reducing the amount of genes for training the model. Very similar results were obtained compared with the original setup, concluding that our approach also works for smaller training sets (Supplementary Figs 4–6).

#### Comparison with GeneMANIA

**Figure 3:**
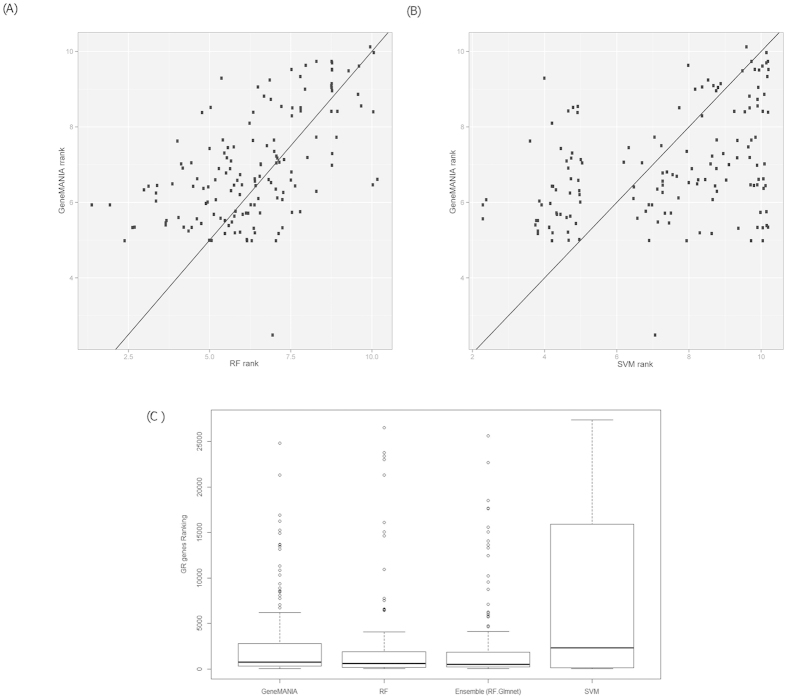
Comparing both the RF Model and the SVM Model to GeneMANIA. Pairwise comparison between (**A**) GeneMANIA and Random Forest (RF), and between (**B**) GeneMANIA and Support Vector Machines (SVM). Each dot represents a *GR* gene, and its coordinates correspond to the ranks assigned by the different methods (ranks are displayed on a logarithmic scale). (**C**) Comparison of the three best prioritization approaches to the state-of-the-art method GeneMANIA.

In order to compare our results with a state-of-the-art gene prioritization tool, we ran GeneMANIA in the same LOOCV setting on our set of *GR* genes. Figure 3C compares GeneMANIA with the best model-based prioritization techniques, and a detailed overview of all ranking criteria is shown in the bottom part of Table 3. While the SVM model clearly outperformed all others only in terms of first quartile results, the RF model markedly outperformed GeneMANIA in terms of both first quartile and median rank. The difference between the RF model and GeneMANIA was significant at a 95% confidence level (Mann-Whitney test, P-value = 0.014).

A detailed comparison of the ranking differences of individual genes is shown using dot plots in Fig. 3, comparing both the RF model and the SVM model to GeneMANIA. Figure 3A shows that most points are plotted above the diagonal, indicating that GeneMANIA in general ranks genes further away than RF, while Fig. 3B shows that clearly two subsets of genes can be distinguished. The top-ranked genes by SVM were all ranked at a worse position by GeneMANIA, while for the remaining genes the situation was reversed.

#### Network importance

To assess the importance of each sub-network for the prioritization, we explored the impact of leaving out each sub-network, and compared the prioritization results without the sub-network to the original approach using all sub-networks integrated. We could observe that removing a single sub-network had only little impact on the prioritization results (Supplementary Figs 7,8), meaning that model-based approaches are clearly robust to the inclusion of noise. Apart from the Naïve Bayes classifier, which benefits from leaving out the PCC sub-network, we saw that the results of most other techniques remained stable when removing individual sub-networks. For the best performing methods, such as SVM, Glmnet and RF, combining sub-networks improved the performance. Here, the inclusion of both the PCC and the GeneMANIA sub-networks appeared to be beneficial for the predictive performance.

### Novel genes predicted to be involved in leaf growth

To obtain the final prediction (ranking) of potential GRs, we used all *GR* genes to train the models, and further limited our analysis to the best model-based prioritization techniques. To this end, we combined all top 200-ranked genes from the model-based methods for which at least 25% of the top 200-ranked genes are encoding known GRs. As a result, only the RF, SVM and LDA models passed this criterion, and genes were sorted by aggregating their ranks for these methods. Because the SVM model performed best in terms of first quartile (the top of the ranking), it could be argued that one should only investigate the results of this single method. However, here, we chose the alternative of having a more robust prediction by inspecting the genes that were ranked consistently high by the best methods.

Among the 100 top 200-ranked genes obtained in this way, a remarkably high number of 57 genes turned out to be known *GR* genes, while 43 were newly predicted genes (Supplementary Table S2). Many of these novel genes have been reported to play some role in leaf growth and development. Out of the 10 most highly ranked novel genes, seven encode important transcription factors that can be linked to growth of leaves or leaf-like structures such as petals and sepals (Table 4). These include the cytokinin response factor *CRF1*, important for cell proliferation during leaf development^25^; *AP-1* (also known as *AGAMOUS-LIKE 7, AGL7*), important for floral meristem and sepal identity^26^; *EGL3* (also known as *MYC-2*), known to be involved in leaf trichome development^27^; *ZFP8*, encoding a zinc finger C2H2 transcription factor involved in the regulation of the fate of leaf epidermal cells^28^; *RGA*, encoding a DELLA protein, known to repress cell proliferation^29^; *TCP15*, a class I TCP gene that modulates cell proliferation in developing leaf blades^30^,^31^ and *YAB3*, encoding a YABBY domain transcription factor that regulates abaxial patterning and growth of lateral organs such a leaves^32^. All these genes are highly ranked in the top 100 lists of the best methods (Supplementary Table S2).

Further down the list, many other (novel) genes/proteins could be linked to leaf growth and development. These include the DELLA protein GAI known to be involved in plant growth^29^; DAWDLE (DDL), an RNA-binding protein that interacts with DCL1 and is involved in miRNA biogenesis and whose mutants result in defective floral organs and smaller leaves^33^; AT5G51190, encoding a member of the ethylene response factor family; AT5G66940, encoding an OBP-1 homolog; AS1 (ASYMMETRIC LEAVES 1), a MYB-like transcription factor with a role in specification of leaf cell identity^34^; HBI1, a basic helix-loop-helix (bHLH) protein involved in the regulation of cell elongation^35^; *ATHB-14* (also known as *PHB*), which encodes a Homeo-Domain-ZIP transcription factor of which dominant *PHB* mutations cause transformation of abaxial leaf fates into adaxial leaf fates^36^, and the microRNA *Mir160* that targets several AUXIN RESPONSE FACTOR (ARF) family members^37^. Also the other novel *GR* genes were highly enriched for transcription factors. In addition, the list of novel *GR* genes contained two genes encoding protein kinases, *ATMMK2* and *ER*, further supporting their regulatory role in plant growth.

In conclusion, the above results convincingly demonstrate the potential of network-based gene prioritization techniques using machine learning approaches for the identification of regulators of biological processes.

### Extension to other important crops

Gene prioritization techniques have a great potential to enhance the likelihood that selected genes have an important role in the process under examination and thus will rapidly gain interest for crop improvement. Here, we show that the genes prioritized for leaf growth are indeed strongly enriched for known leaf growth regulators that were not in the set of a priori selected genes. The entire data set needs to be validated further by functional analysis. Processes such as growth regulation are well conserved and there are many examples of genes originally found to affect growth in Arabidopsis that also function in a similar manner in other crops^38^,^39^. This renders the prioritization techniques developed here of high importance for crop improvement by considering that the genomes of many crops have now been sequenced, and next generation RNA sequencing technologies allow for determining the transcriptomes of any tissue in virtually any species. In addition, large-scale phenotyping platforms – in particular in rice – made it possible to identify central regulators of yield-related processes that can be used as ‘seeds’ for the prioritization techniques.

Recently, it also became clear that the combination of growth-promoting genes most often leads to additive or synergistic effects on growth^40^ and, although still subject to experimental testing, we postulate that genes highly ranked in the prioritization list have a higher potential to be combined than low-ranked genes.

## Methods

### An integrated network for Arabidopsis growth regulation

To construct the integrated network, we followed an entirely quantitative approach, in which individual networks were joined by adding the entity values of each network’s adjacency matrix. To this end, a large adjacency matrix, in which columns and rows represent gene identifiers from all individual sub-networks, and entries show the number of edges between each pair of genes, was made. The number of edges between each pair of nodes was determined by summing the number of edges in all seven sub-networks. As a result, any pair of nodes is connected by at most seven edges. The rationale for keeping all edges between a fixed pair of genes was that links from different data sources provide additional evidence for a relationship between two genes.

By looking at network topology properties, one can explore the integrated network. For these properties, multiple edges between the same pair of genes only affected the degree, Kleinberg’s hub and authority scores, for which we explicitly took multiple edges into account. For all other properties, i.e. those related to the shortest path concept (e.g. betweenness, closeness and shortest path to other nodes), the presence of multiple edges between a gene pair did not influence the score. In addition, all data sources were treated similarly, and no preferences or weights on the edges were introduced.

#### AGRIS regulatory sub-network

The Arabidopsis Gene Regulatory Information Server (AGRIS) supplies a resource for gene regulatory studies in Arabidopsis thaliana. A component of AGRIS, the Arabidopsis thaliana regulatory network database (AtRegNet) consists of transcription factors and their direct target genes only^18^. The AtRegNet database was converted into a network, keeping for each transcription factor its direct target genes.

#### MaMut genetic modification design sub-network

This sub-network was extracted from the “genetic modification data set” of CORNET, a publicly available database on gene associations in plants^19^. This network contains information about differentially expressed genes when comparing wild-type plants to transgenic plants. Links in this network represent genes that are either up- or down-regulated when knocking out one or more transcription factors. These differentially expressed genes are assumed to be the target (either direct or indirect) of the transcription factor that was knocked out.

#### PPI sub-network

The PPI sub-network was extracted from CORNET, and includes predicted, as well as experimentally identified, PPIs in Arabidopsis from different sources. Some of these interactions were derived from the original AraNet network^14^.

#### GeneMANIA sub-network

The GeneMANIA sub-network represents a combination of different publicly available data sets, collected from a variety of databases. These include co-expression data, co-localization data, genetic interactions, physical interactions, shared protein domains and predicted interactions, all combined into a single network. A detailed overview of all networks used by GeneMANIA can be found on the GeneMANIA website (http://genemania.org/).

#### GENIE3 predicted regulatory sub-network

In order to construct computationally predicted transcriptional regulatory networks, we used the GENIE3 algorithm, which achieved the best performance on the DREAM5 network inference challenge^21^. GENIE3 was run on the CORNET “leaf” compendium, which consists of 212 different conditions and time points, and is a collection of publicly available and in-house generated expression data, which has been pre-processed through a well-defined normalization and quality control pipeline developed for the CORNET database. The result of this analysis is a list of predicted transcription factor-target relations and an associated confidence score. Using a cutoff of 0.02, we only used the most confident associations to build the network.

#### Co-expression sub-network using Pearson correlation coefficient (PCC)

Using the “leaf” compendium from the CORNET database, we calculated a co-expression network by calculating the Pearson correlation coefficient between the expression patterns of all genes. As the resulting network was huge, we only kept the 5% most significant gene pairs, corresponding to the correlations that had at least an absolute value of about 0.8, thus keeping the most correlated or anti-correlated genes.

#### Text mining sub-network

A network of gene-gene associations predicted using text mining was extracted from the EVEX text mining resource^22^. Text mining was built on top of PubMed abstracts and PubMed Central full text articles, covering over 40 million bio-molecular events among more than 76 million automatically extracted gene/protein name mentions. The text mining data further has been enriched with gene identifiers and gene families from Ensemble and HomoloGene, providing homology-based event generalizations. In this work, we only used gene-gene associations that were annotated as belonging to Arabidopsis genes.

### Arabidopsis growth regulators

As input for the prioritization approach, we started from a seed set of genes that is known to be involved in leaf growth, and that consists of two subsets. The first set of 57 genes (Intrinsic Yield Genes) was based on published effects on leaf size when mutated or ectopically expressed (www.yieldbooster.com)^6^,^7^. The second set consisted of putative growth-related genes selected from genome-wide transcript profiling of micro-dissected developing leaves, harvested and profiled daily at six time points from day 8 (when all cells are proliferating) until day 13 (when most leaf cells start to expand) after stratification^23^. For each time point, three biological replicates were measured. 9,585 genes that were differentially expressed between at least two time points were identified with a moderated F-test and a corrected P-value of 0.05. Pairwise comparisons between time points were tested with moderated t-statistics and eBayes method as implemented in Limma, P-values were corrected for multiple testing^41^.

Subsequently, we further filtered this set of genes and only considered genes showing a difference in expression of at least twofold at two time points (pairwise comparison between all time points), and only kept genes that showed evidence of transcriptional activity. Eventually, four genes were listed as transcription factors based on AGRIS, and 94 genes were retained based on Gene Ontology terms (GO:0003677 (DNA binding), GO:0006355 (regulation of transcription) and GO:0003700 (transcription factor activity), GO:0005524 (ATP binding), GO:0003713 (transcription coactivator activity), GO:0003676 (nucleic acid binding), GO:0008270 (zinc ion binding), GO:0005515 (protein binding)).

In total we thus obtained a list of 98 genes that not only showed a significant expression change during leaf development, but that were also listed as regulators and transcription factors, showing a high level of evidence to consider them as genes involved in regulation of leaf developmental processes.

### Connectivity patterns

In graph theory, many mathematical properties can be extracted from a graph or network. We used these properties to a) characterize our set of known *GR* genes, and b) to derive new graph-based prioritization methods. These network patterns could be divided into two main categories: general topological patterns that can be extracted from any network, and similarity-based patterns that can be defined on a network with regard to a set of genes of interest (e.g. Arabidopsis *GR* genes in our case).

### General topological patterns

General topological properties of a sub-network capture connectivity properties of each gene in the sub-network. These include the degree of each gene (the number of connections it has to other genes), the betweenness centrality (a measure of how central the gene is when passing through it on a path connecting two other genes), the closeness (a measure that represents how fast information from the current gene spreads over the network), and Kleinberg’s hub and authority scores, which are based on the principal eigenvector of the adjacency matrix of the sub-network. A detailed overview of these general topological properties, as well as formulas to calculate these properties, can be found in the Supplementary Information.

### Similarity-based patterns

Similarity-based patterns are defined with regard to a specific subset of genes (nodes) in the network. They can be divided into two classes: topology-based and Gene Ontology (GO)-based similarity measures.

The first class measures the similarity between two genes, or more generally two groups of genes using network-based patterns. For the purpose of this study, we calculated these properties between a chosen gene (query gene) and a seed set of genes of interest S (e.g. Arabidopsis *GR* genes). The properties we took into account included the number of direct connections with S, the number of shared genes with S, the Jaccard similarity index, the Dice similarity index, the inverse log-weighted index and the shortest path to S. A detailed explanation can be found in the Supplemental Information.

The second class of similarity-based patterns uses a combination of GO terms and topological properties. Two types of measures were explored: an approach based on GO term overlap and an approach based on GO enrichment. For the approach based on GO term overlap, we defined three term overlap similarity measures: term overlap between neighbors of a query gene and the seed genes, term overlap between the query gene itself and the seed genes, and finally the combination of both. The magnitude of the term overlap was measured by the Jaccard similarity coefficient. For the approach based on GO enrichment, we followed the approach proposed in Rahmani, *et al*.^42^. We counted relevant GO terms for S, and selected the top ten statistically most overrepresented terms. Next, for these terms a two-way table was constructed using the frequency of the terms in the seed genes as well as the query gene and its neighbors.

The reason for including neighbors was the fact that GO annotations of proteins can often be predicted well based on the GO annotations of their neighbors^43^,^44^. The P-value of a Fisher exact test comparing the frequency of terms in the two groups was then used as a similarity measure. The higher the P-value, the more similar the representation of GO terms between the two groups will be. The same procedure was then also applied for the top five and the first GO terms.

### Machine learning models

**Figure 4:**
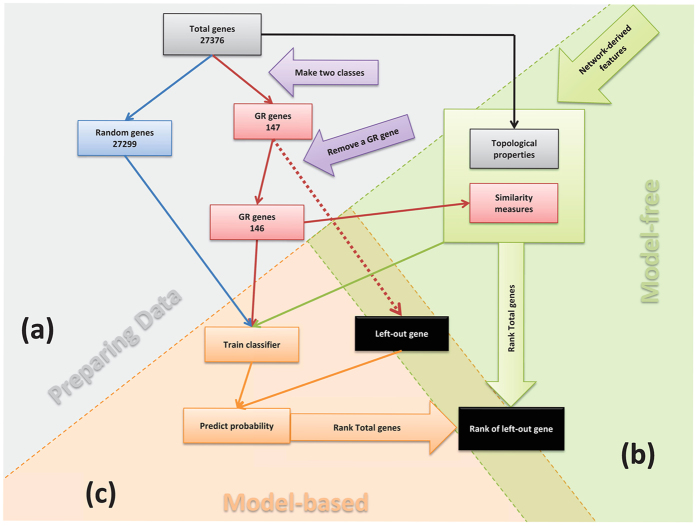
Leave-One-Out Cross-Validation (LOOCV) Workflow. Graphical overview of the workflow when assessing the predictive performance of methods using the LOOCV setup. (**a**) Preparing data and making two classes to feed the computational parts, (**b**) extract multiple types of features from the network, and (**c**) combining network-derived features with machine learning models resulting in model-based prioritization approaches.

In the model-based approach for prioritization, we used machine learning models to define the relationship between the network properties and the genes in the seed set S. We used a two-class classification approach for the prioritization problem, where the genes in S were assumed to constitute the “positive” class, and the remaining genes represented the “negative and unknown” class. In our case, the positive class was the set of 147 genes involved in leaf growth, and the negative/unknown class consisted of the remaining 27,229 genes of the Arabidopsis genome. To evaluate the prioritization performance of the different methods, a leave-one-out cross-validation (LOOCV) setup was used, which is standard in the prioritization field^10^. In this setup, one of the *GR* genes was removed from the list of known *GR* genes, and subsequently all genes in the network were ranked, the most top-ranked genes corresponding to the most likely *GR* genes. The position of the left-out gene could then be recorded, and this procedure was subsequently repeated for all *GR* genes. Performance statistics, such as the median or average rank of all *GR* genes could then be used to evaluate prioritization performance. A general overview of the followed procedure is shown in Fig. 4.

In this work, we used six machine learning models that are commonly used in the literature: Naïve Bayes (NB), Linear discriminant analysis (LDA), Support Vector Machines (SVM), Lasso and elastic-net regularized generalized linear models (Glmnet), Random Forest (RF), and Generalized Boosted Regression Models (GBM). More information about the parameter settings and implementation can be found in the Supplemental Information. For each of these models, the following set of 35 features was used: the general topological patterns (5 features), the topology-based similarity patterns (6 features), and 24 features derived from the GO-based similarity measures. The six GO-based similarity measures were calculated four times: once for complete GO terms, once just for the “biological process” category, once for the “cellular component” category and finally once for the “molecular function” category. Splitting these categories over different features would allow the classification models to weigh them differently according to their information content (Supplementary Fig. S9).

### GeneMANIA

GeneMANIA uses a Gaussian field label propagation algorithm for binary classification, taking as input an association network, a list of nodes with positive labels, possibly a list of nodes with negative labels, and initial label bias values. In our experiments, only a list of positive labels (the *GR* genes) was available. For gene prioritization, each gene was associated with a graph node and nodes representing *GR* genes (that is, positive genes) were assigned an initial label bias value of +1, whereas all other, unlabelled genes were assigned a value k = n +/n, where n+ denotes the number of positive genes, and n denotes the total number of genes. The label propagation algorithms then assigned a discriminant value to each node by letting the initial label bias of nodes propagate through the association network to nearby nodes. These discriminant values were then used to prioritize (rank) all genes. Determining the discriminant values can be done by solving a quadratic programming problem^16^.

## Additional Information

**How to cite this article**: Sabaghian, E. *et al*. An integrated network of Arabidopsis growth regulators and its use for gene prioritization. *Sci. Rep.* **5**, 17617; doi: 10.1038/srep17617 (2015).

## Supplementary Material

Supplementary Information

## Figures and Tables

**Table 1:** Global Topology of Each Sub-Network.

Sub-network	# Edges	# Nodes	Density	Clusteringcoefficient	Diameter	Averagepathlength
AGRIS	13,033	9,443	3.48E-05	0.001	7	2.68
MaMut	142,299	17,147	3.80E-04	0.003	6	2.61
PPI	1,147,589	22,372	3.06E-03	0.267	18	3.32
PCC	12,526,356	11,763	3.34E-02	0.745	15	2.47
GENIE3	281,327	21,503	7.51E-04	0.021	5	3.05
GeneMANIA	10,244,303	24,815	2.73E-02	0.135	6	2.09
EVEX	6,499	2,335	1.73E-05	0.170	15	5.49
**Total**	**24,361,406**	**27,376**	**6.50E-02**	**0.553**	**11**	**2.13**

Network statistics of the different sub-networks as well as the total, integrated network.

**Table 2:** Network Contribution.

Sub-network	Local *GR* gene network			Local *GR* gene network + first neighbors		
	Edgenumber	PercentTotal	PercentIndividual	Edge number	PercentTotal	PercentIndividual
**AGRIS**	4	0.11%	0.03%	448	0.09%	3.44%
**MaMut**	65	1.77%	0.05%	6338	1.30%	4.45%
**PPI**	85	2.32%	0.01%	9421	1.93%	0.82%
**PCC**	1952	53.25%	0.02%	301262	61.61%	2.41%
**GENIE3**	360	9.82%	0.13%	29474	6.03%	10.48%
**GeneMANIA**	1163	31.72%	0.01%	141537	28.95%	1.38%
**EVEX**	37	1.01%	0.57%	491	0.10%	7.56%
**Total**	3666	Nodes = 147		488971	Nodes = 22078	

Network contribution of the different sub-networks constituting the integrated network, both for the sub-network network connecting the *GR* genes (left column) as well as the extended network containing GRs and their first neighbors in the integrated network (right column).

**Table 3:** Performance Statistics for GR Prioritization Using Model-Based Approaches.

Model		Min	Firstquartile	Median	Thirdquartile	Max	IQR	Percentage of *GR*genes within firstquartile
NaiveBayes	Without GO	3	2676	6061	7831	26557	5154	1.3%
	With GO	13	629	2480	6147	25452	5518	5.9%
SVM	Without GO	13	813	5054	11789	27169	10976	4.5%
	With GO	10	127	2313	15930	27375	15803	29%
LDA	Without GO	13	965	2645	6362	27255	5397	3.8%
	With GO	12	481	1551	4600	27255	4119	7.7%
Glmnet	Without GO	3	753	2616	4974	26276	4220	4.9%
	With GO	5	279	885	2442	25521	2163	13.3%
GBM	Without GO	2	788	3966	7174	26560	6386	4.5%
	With GO	11	899	2260	5986	26542	5086	4.1%
RF	Without GO	3	415	2103	6178	26505	5769	8.9%
	With GO	4	181	589	1893	26544	1711	20%
RF + Glmnet		4	229	520	1832	25652	1603	16.2%
GeneMANIA		12	312	738	2781	24815	2468	11.9%

Overview of the different ranking statistics, including the minimum and maximum rank, the first and third quartile rank, the median rank, the inter-quartile range (IQR) and the percentage of *GR* genes retrieved within the first quartile.

**Table 4:** Interpretation of Top Ten Novel *Genes Encoding Growth Regulators (GRs)*.

Rank	Gene	AT code	Evidence
1	CRF1	AT4G11140	Cytokinin response factor involved in cytokinin signaling. Cytokinins stimulate cell proliferation during leaf development.
2	AP1	AT1G69120	Floral homeotic gene encoding a MADS domain protein. Specifies floral meristem and sepal identity. Required for the transcriptional activation of AGAMOUS. Interacts with LEAFY.
3	EGL3	AT1G63650	Transcription factor known to be involved in leaf trichome development. Also known as MYC-2. Mutants have reduced trichomes.
4	RDO2	AT2G38560	Encodes RNA polymerase II transcript elongation factor TFIIS. Mutant plants display essentially normal development, but they flower slightly earlier than the wild type and show clearly reduced seed dormancy.
5	ZFP8	AT2G41940	Encodes a C2H2 transcription factor involved in the regulation of the fate of leaf epidermal cells. Controls shoot maturation and epidermal cell fate by integrating gibberellins (GAs) and cytokinin signaling in Arabidopsis.
6	RGA	AT2G01570	Repressor of GA. Member of the DELLA regulatory family. Putative transcriptional regulator repressing the gibberellin response and integration of phytohormone signalling. DELLAs repress cell proliferation and expansion that drives plant growth.
7	TCP15	AT1G69690	Involved in the regulation of endoreduplication. Belongs to class I TCP genes. Modulates cell proliferation in developing leaf blades.
8	ICE1	AT3G26744	Encodes a MYC-like bHLH transcriptional activator that binds specifically to the MYC recognition sequences in the CBF3 promoter. Mutants are defective in cold-regulated gene expression.
9	YAB3	AT4G00180	YABBY gene family member, likely has transcription factor activity, and is involved in specifying the abaxial cell fate of leaves.
10	ATB2	AT1G60710	Probably encodes an aldo-keto reductase.

The top 10 novel *GR* genes identified by our novel approach, and a literature summary highlighting their potential role in leaf growth.
