# Supplementary material for: An integrated network of Arabidopsis growth regulators and its use for gene prioritization
Source: Sci Rep. 2015 Dec 1;5:17617. doi: 10.1038/srep17617 (PMC4664945; doi:10.1038/srep17617)
Supplement: Supplementary Information [file srep17617-s1.pdf]

Supplementary Material

for

An integrated network of Arabidopsis growth regulators and its use for prioritization

Ehsan Sabaghian, Zuzanna Drebert, Dirk Inzé, Yvan Saeys

Supplementary Figures

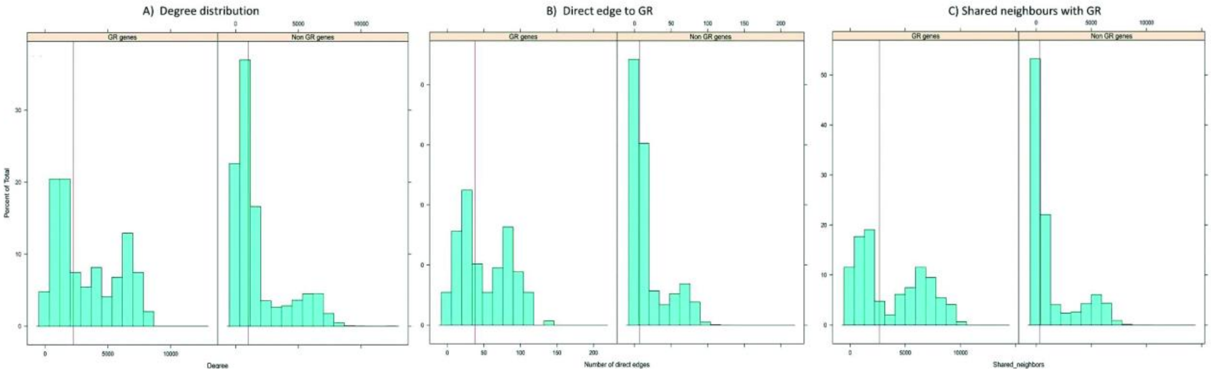

Figure S1 - General topological properties distribution

A) Degree distribution of growth regulators versus non growth regulators. The median degree for GR genes is 2259 (black line), while this value for non-GR genes is 1023.

B) Distribution of the number of direct edges to growth regulators for growth regulators themselves, and non-growth regulators. The median for GR genes is 38, while for non GR genes it is 7.

C) Distribution of the number of shared neighbours for growth regulators, and non growth regulators. The median for GR genes is 2673, while it is 335 for non GR genes.

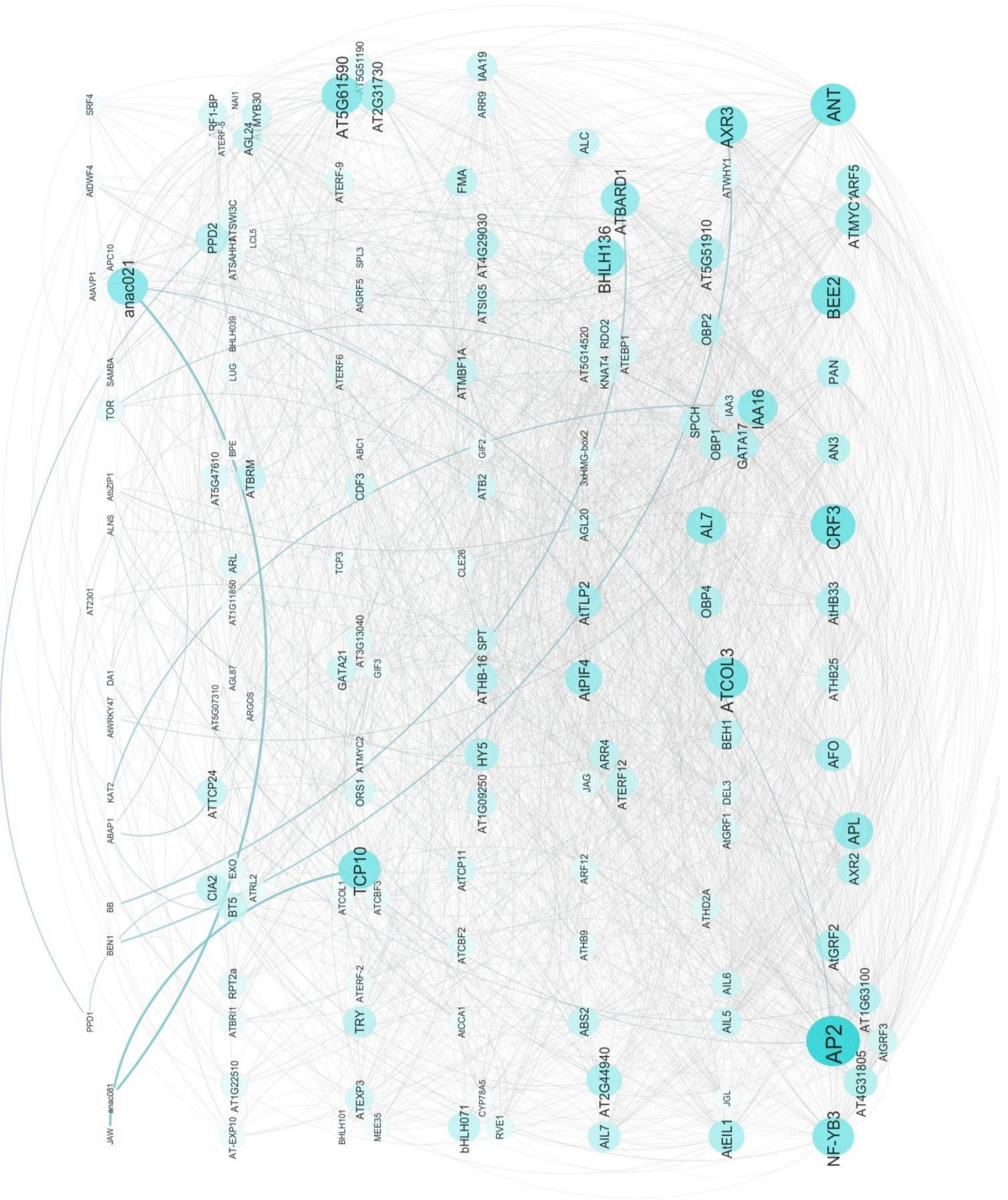

Figure S2 - Edge and node betweenness for the local network of growth regulators. A bigger font size of the nodes (genes) corresponds to a more prominent role in connecting subparts of the network, while an increased thickness of the edges corresponds to the edge betweenness, illustrating paths that are taken more frequently when connecting nodes. Genes are again organized into layers based on their degree.

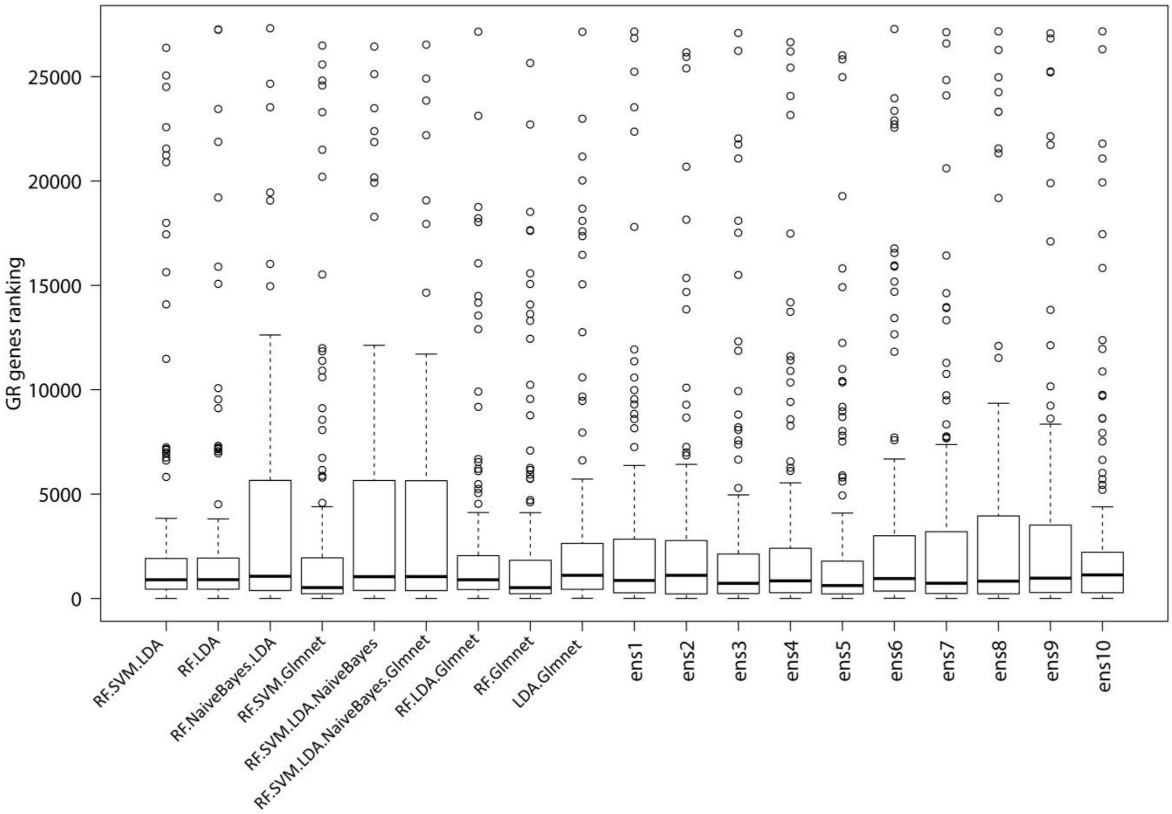

Figure S3 - GR ranks in the ensemble approach

Crossvalidation setup

In addition to the leave-one-out crossvalidation (LOOCV) setup, we also explored the effect of using less GR genes for training the model, using a 10-fold crossvalidation setup. Figure S4 shows a comparison between the LOOCV setup and the 10-fold crossvalidation for the model-based approaches. Similar graphs are shown for the comparison of the best models to GeneMANIA (Figure S5), and the best ensemble models (Figure S6).

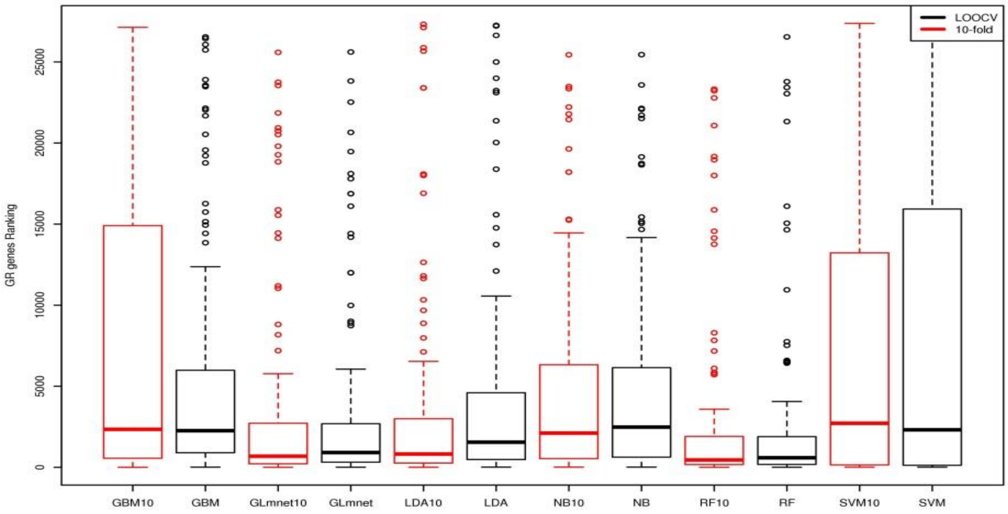

Figure S4 - Comparison between leave-one-out crossvalidation and 10-fold crossvalidation for the model-based approaches.

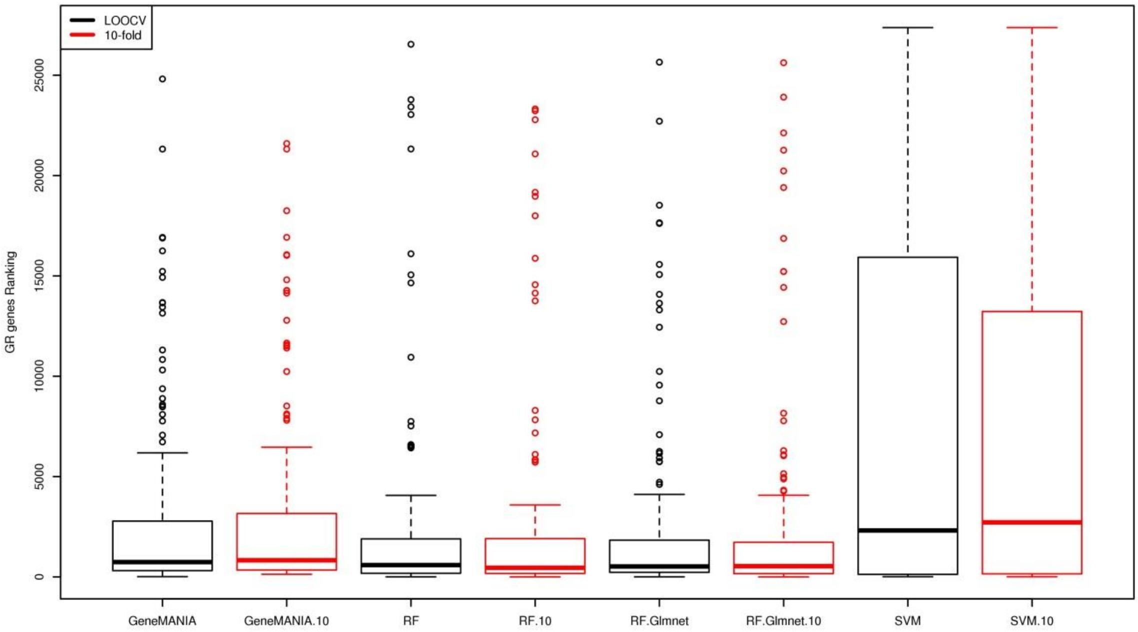

Figure S5 - Comparison between the leave-one-out crossvalidation and the 10-fold crossvalidation for the best model-based prioritization models.

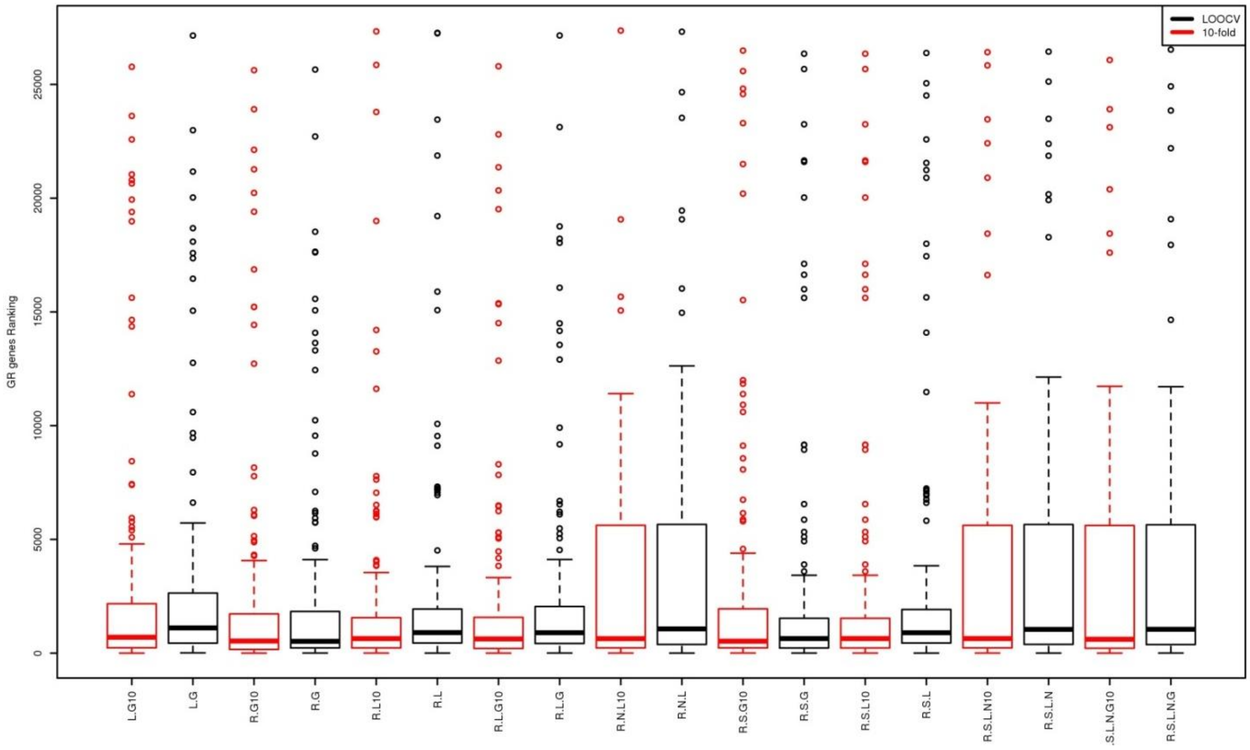

Figure S6 - Comparison between the LOOCV and the 10-fold crossvalidation for the best ensemble models.

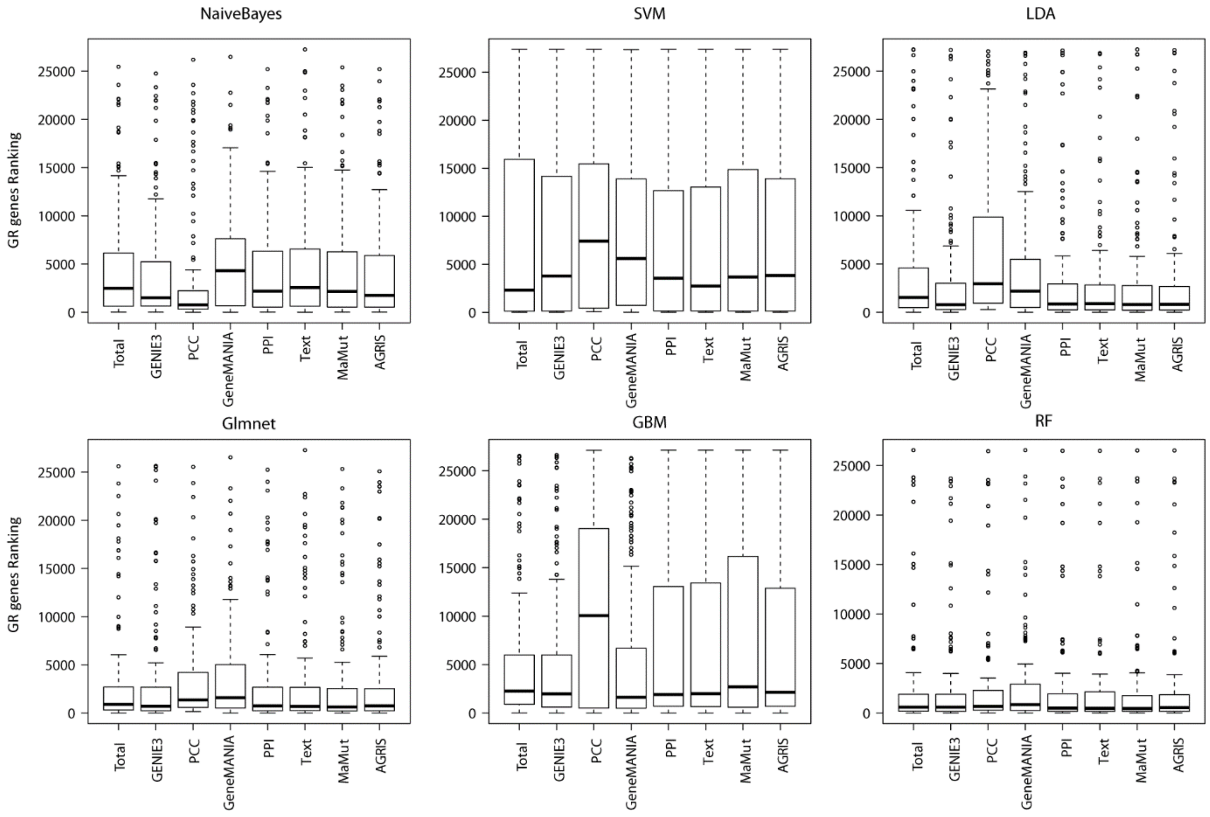

Figure S7 - Network importance for the model-based approaches

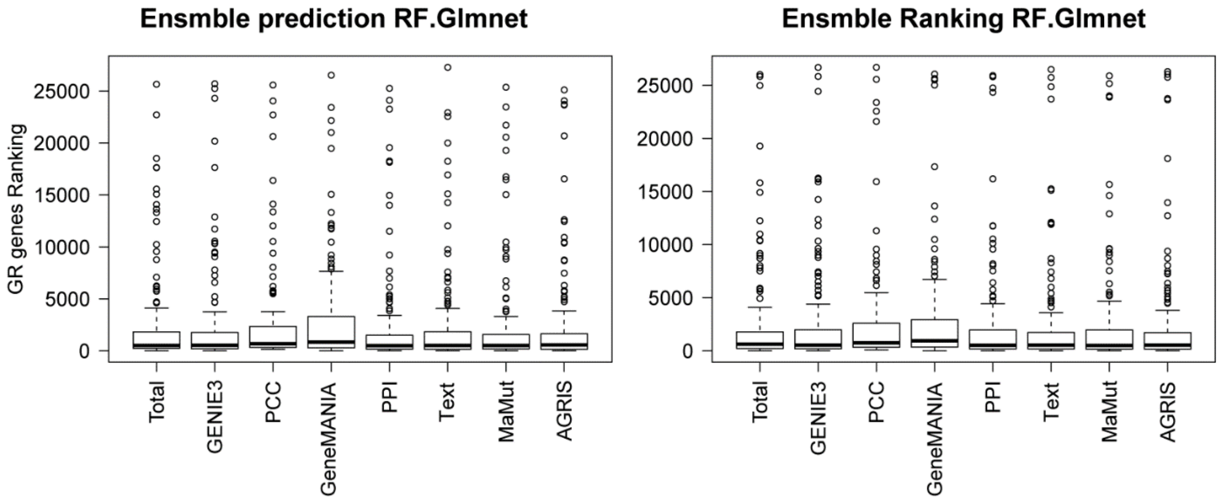

Figure S8 - Network importance for the best ensemble method

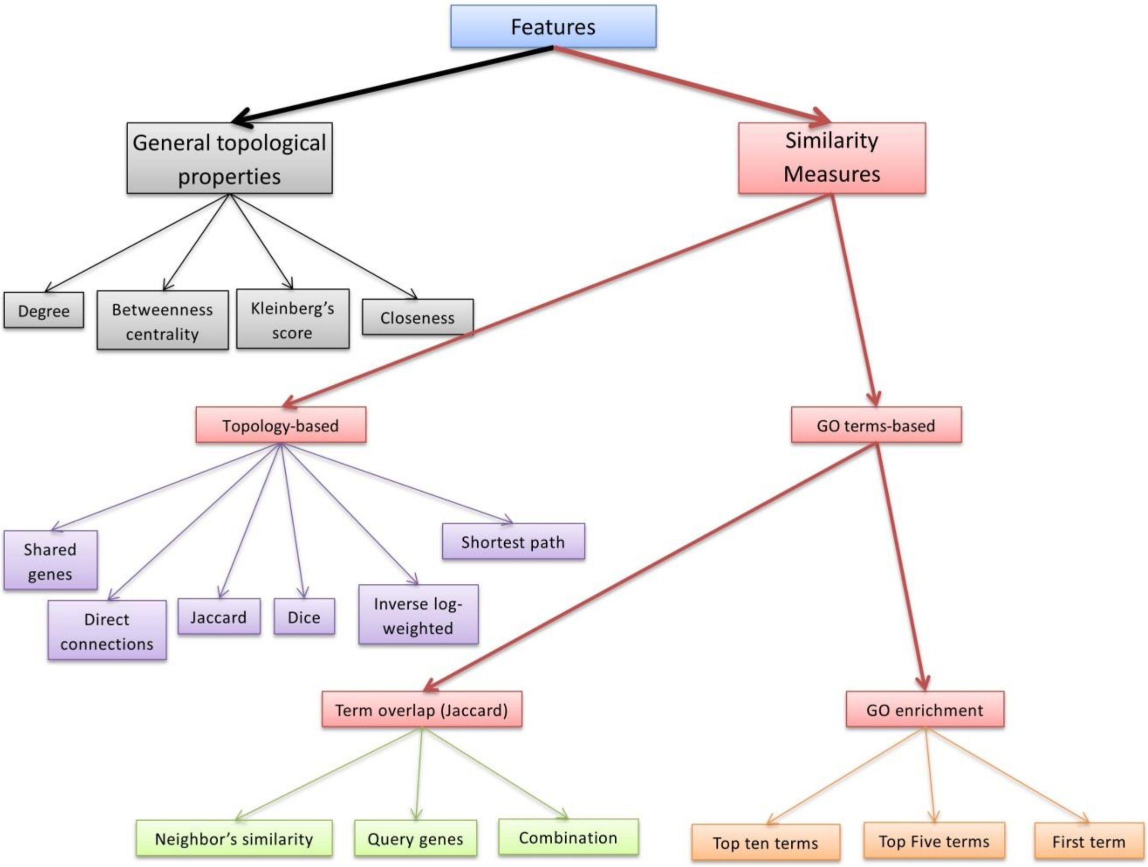

Figure S9- Network derived features

Supplementary methods

Arabidopsis growth regulators

Table S1 gives an overview of all genes that were used as input for the prioritization algorithm. These include both the Intrinsic Yield Genes (IYG) as well as the growth regulators that were identified through a microarray experiment of developing early leaves (GR).

AT codes	Type	Name
AT1G01160	IYG	GIF2
AT1G07640	IYG	OBP2
AT1G13710	IYG	CYP78A5
AT1G15690	IYG	ATAVP3
AT1G19270	IYG	DA1
AT1G21700	IYG	ATSWI3C
AT1G26770	IYG	AT-EXP10
AT1G30210	IYG	ATTCP24
AT1G32310	IYG	SAMBA
AT1G50030	IYG	TOR
AT1G53230	IYG	TCP3
AT1G56010	IYG	anac021
AT1G59640	IYG	BPE
AT1G69970	IYG	CLE26
AT1G75240	IYG-GR	AtHB33
AT2G18290	IYG	APC10
AT2G22840	IYG-GR	AtGRF1
AT2G31070	IYG	TCP10
AT2G33150	IYG	KAT2
AT2G37640	IYG	ATEXP3
AT2G41230	IYG	ORS1
AT2G44080	IYG	ARL
AT2G45400	IYG	BEN1
AT2G46020	IYG	ATBRM
AT3G13065	IYG	SRF4
AT3G13960	IYG-GR	AtGRF5
AT3G15030	IYG-GR	MEE35
AT3G50660	IYG	CLM
AT3G51800	IYG	ATEBP1
AT3G59900	IYG	ARGOS
AT3G63530	IYG	BB
AT4G00850	IYG	GIF3
AT4G08950	IYG	EXO
AT4G13940	IYG	ATSAHH1
AT4G14713	IYG	PPD1
AT4G17490	IYG-GR	ATERF6
AT4G23713	IYG	JAW
AT4G25420	IYG	AT2301
AT4G29040	IYG	RPT2a
AT4G32551	IYG	LUG
AT4G36930	IYG	SPT
AT2G27050	GR	AtEIL1
AT2G31730	GR	-
AT2G33810	GR	SPL3
AT2G36080	GR	ABS2
AT2G36400	GR	AtGRF3
AT2G38560	GR	TFIIS
AT2G42680	GR	ATMBF1A
AT2G43010	GR	PIF4
AT2G44940	GR	-
AT2G45190	GR	AFO
AT2G45660	GR	AGL20
AT2G46830	GR	CCA1

AT codes	Type	Name
AT4G37740	IYG-GR	AtGRF2
AT4G37750	IYG-GR	ANT
AT4G39400	IYG	ATBRI1
AT4G40060	IYG	ATHB-16
AT5G07310	IYG	-
AT5G08790	IYG	anac081
AT5G10510	IYG	AIL6
AT5G13060	IYG	ABAP1
AT5G18010	IYG	SAUR19
AT5G28640	IYG-GR	AN3
AT5G47230	IYG	ATERF-5
AT5G53290	IYG	CRF3
AT5G57390	IYG	AIL5
AT5G58220	IYG	TTL
AT5G62000	IYG	ARF1-BP
AT5G65510	IYG	AIL7
AT1G04020	GR	ATBARD1
AT1G04240	GR	IAA3
AT1G04250	GR	AXR3
AT1G08540	GR	ABC1
AT1G09250	GR	AIF4
AT1G10470	GR	ARR4
AT1G11850	GR	-
AT1G13400	GR	JGL
AT1G14410	GR	ATWHY1
AT1G14510	GR	AL7
AT1G19850	GR	ARF5
AT1G22510	GR	-
AT1G22590	GR	AGL87
AT1G28360	GR	ATERF12
AT1G30490	GR	ATHB9
AT1G32640	GR	ATMYC2
AT1G34310	GR	ARF12
AT1G63100	GR	-
AT1G68480	GR	JAG
AT1G68640	GR	PAN
AT1G79430	GR	APL
AT2G18280	GR	AtTLP2
AT2G21650	GR	ATRL2
AT2G22770	GR	NAI1
AT2G24790	GR	ATCOL3
AT5G14520	GR	-
AT5G15850	GR	ATCOL1
AT5G17300	GR	RVE1
AT5G24120	GR	ATSIG5
AT5G39860	GR	BHLH136
AT5G44210	GR	ATERF-9
AT5G46690	GR	bHLH071
AT5G47220	GR	ATERF-2
AT5G47610	GR	-
AT5G49450	GR	AtbZIP1
AT5G51190	GR	-
AT5G51910	GR	-

AT codes	Type	Name
AT4G37610	GR	BT5
AT5G04150	GR	BHLH101
AT5G08330	GR	ATTCP11
AT5G11060	GR	KNAT4
AT5G11260	GR	HY5
AT3G04730	GR	IAA16
AT3G09600	GR	LCL5
AT3G13040	GR	-
AT3G15540	GR	IAA19
AT3G16870	GR	GATA17
AT3G23050	GR	AXR2
AT3G24140	GR	FMA
AT3G28910	GR	ATMYB30
AT3G44750	GR	ATHD2A
AT3G47500	GR	CDF3
AT3G50410	GR	OBP1
AT3G50750	GR	BEH1
AT3G56980	GR	BHLH039
AT3G57040	GR	ARR9
AT4G00480	GR	ATMYC1
AT4G01720	GR	AtWRKY47
AT4G14540	GR	NF-YB3
AT5G53210	GR	SPCH
AT5G56860	GR	GATA21
AT5G57180	GR	CIA2
AT5G60850	GR	OBP4
AT5G61590	GR	-
AT5G65410	GR	ATHB25
AT5G67110	GR	ALC
AT4G14720	GR	PPD2
AT4G23800	GR	3XHMG-BOX2
AT4G24540	GR	AGL24
AT4G25470	GR	ATCBF2
AT4G25480	GR	ATCBF3
AT4G29030	GR	-
AT4G31805	GR	POLAR
AT4G34590	GR	ATB2
AT4G36540	GR	BEE2
AT4G36920	GR	AP2
AT3G01330	GR	DEL3
AT5G53200	GR	TRY

Table S1 - List of GR genes, detailing for each gene the AT-code, whether it is an Intrinsic Yield Gene (IYG) or a growth regulator identified by microarray analysis (GR), and the TAIR gene symbol.

Rank	Name	Freq in top 21 model-based approach
1	AFO	21
2	AGL7	21
3	AIL5	21
4	AN3	21
5	ANT	21
6	AP2	21
7	ARF5	21
8	TCP15	21
9	ATB2	21
10	GLK1	21
11	GRF2	21
12	GRF3	21
13	HB-2	21
14	HB25	21
15	HB33	21
16	MYB108	21
17	MYC1	21
18	MYC2	21
19	MYC-2	21
20	AXR2	21
21	BEE2	21
22	CCA1	21
23	CRF1	21
24	CRF3	21
25	DDL	21
26	FMA	21
27	GATA21	21
28	HY5	21
29	IAA3	21
30	JAG	21
31	MEE35	21
32	OBP2	21
33	PAN	21
34	PIF4	21
35	RGA	21
36	TCP10	21
37	TCP3	21
38	TFIIS	21
39	YAB3	21
40	ZFP8	21
41	AGL22	20
42	AGL24	20
43	AIL6	20
44	AS1	20
45	AT1G61660	20
46	HBI1	20
47	AT3G06220	20
48	ERF11	20
49	GRF1	20
50	HB-14	20

Rank	Name	Freq in top 21 model-based approach
51	ICE1	20
52	MKK2	20
53	AXR3	20
54	BLH9	20
55	BP	20
56	ERL2	20
57	IAA2	20
58	JGL	20
59	LHW	20
60	LUG	20
61	MIF1	20
62	PIF5	20
63	TOR	20
64	ALC	19
65	AT4G17810	19
66	AT5G66940	19
67	PIN1	19
68	PIN3	19
69	BHLH136	19
70	RVE1	19
71	SPCH	19
72	AGL9	18
73	AHK3	18
74	HB31	18
75	HB-4	18
76	HB51	18
77	LHY	18
78	STO	18
79	TOE3	18
80	AT1G23000	17
81	AT3G17100	17
82	BZIP61	17
83	CPK32	17
84	HB-8	17
85	MYB15	17
86	MYB17	17
87	MYB51	17
88	PARP2	17
89	EDF4	17
90	HUB1	17
91	ROXY1	17
92	RPS2	17
93	RPT2a	17
94	SNZ	17
95	ABI5	16
96	AGL20	16
97	AIL7	16
98	AT2G44940	16
99	GRF5	16
100	-GTL1	16

Table S2 - 100 best-ranked genes. 57 turn out to be known growth regulators (green labeld), while 43 are newly predicted genes.

GO ID	P-value	Odds Ratio	GO term	Ontology
GO:0003700	3.12E-35	5.43	Has sequence-specific DNA binding transcription factor activity	MF
GO:0006355	3.27E-30	4.75	regulation of transcription, DNA-dependent	BP
GO:0003677	1.43E-19	3.87	DNA binding	MF
GO:0048366	7.92E-18	16.07	leaf development	BP
GO:0005515	1.32E-10	3.06	protein binding	MF
GO:0045893	4.38E-10	5.09	positive regulation of transcription, DNA-dependent	BP
GO:0048481	2.25E-09	9.03	ovule development	BP
GO:0009965	3.89E-09	7.81	leaf morphogenesis	BP
GO:0009739	2.03E-07	9.86	response to gibberellin stimulus	BP
GO:0003713	2.91E-07	27.55	transcription coactivator activity	MF

Table S3 - Top ten most overrepresented GO terms in GR genes.

Name	Combination	Type
Ens1	NB+RF+GBM+SVM+LDA+Glmnet	Ranking ensemble
Ens2	NB+RF	Ranking ensemble
Ens3	RF+LDA+Glmnet	Ranking ensemble
Ens4	RF+LDA	Ranking ensemble
Ens5	RF+Glmnet	Ranking ensemble
Ens6	LDA+Glmnet	Ranking ensemble
Ens7	RF+SVM+LDA+Glmnet	Ranking ensemble
Ens8	NB+RF+SVM	Ranking ensemble
Ens9	NB+RF+GBM+SVM	Ranking ensemble
Ens10	NB+RF+LDA+Glmnet	Ranking ensemble
R.S.L	RF+SVM+LDA	Prediction probability ensemble
R.L	RF+LDA	Prediction probability ensemble
R.N.L	RF+NB+LDA	Prediction probability ensemble
R.S.G	RF+SVM+Glmnet	Prediction probability ensemble
R.S.L.N	RF+SVM+LDA+NB	Prediction probability ensemble
R.S.L.N.G	RF+SVM+LDA+NB+Glmnet	Prediction probability ensemble
R.L.G	RF+LDA+Glmnet	Prediction probability ensemble
R.G	RF+Glmnet	Prediction probability ensemble
L.G	LDA+Glmnet	Prediction probability ensemble

Table S4 - Overview of the different ensemble strategies explored in this work.

Quartiles	Min	First Quartile	Median	Third Quartile	Max	IQR	Percentage of GR genes within first quartile
RF.SVM.LDA (R.S.L)	2	446	897	1919	26382	1473	8.3%
RF.LDA (R.L)	2	446	903	1938	27267	1492	8.3%
RF.NaiveBayes.LDA (R.N.L)	2	383	1066	5658	27316	5276	9.7%
RF.SVM.Glmnet (R.S.G)	4	230	525	1948	26486	1719	16.1%
RF.SVM.LDA.NaiveBayes (R.S.L.N)	2	385	1044	5656	26440	5271	9.6%
RF.SVM.LDA.NaiveBayes.Glmnet (R.S.L.N.G)	4	378	1048	5643	26531	5265	9.8%
RF.LDA.Glmnet (R.L.G)	4	426	898	2051	27151	1626	8.7%
RF.Glmnet (R.G)	4	229	520	1832	25652	1603	16.2%
LDA.Glmnet (L.G)	11	439	1109	2641	27147	2202	8.4%
Ens1	5	276	865	2841	27159	2565	13.4%
Ens2	3	221	1109	2772	26161	2552	16.7%
Ens3	4	236	726	2131	27093	1895	15.7%
Ens4	4	279	845	2405	26654	2126	13.3%
Ens5	3	221	623	1791	26020	1570	16.7%
Ens6	10	354	956	3006	27277	2652	10.5%
Ens7	3	242	733	3202	27126	2960	15.3%
Ens8	2	226	828	3956	27166	3730	16.4%
Ens9	2	287	973	3522	27073	3235	12.9%
ens10	6	278	1130	2216	27165	1938	13.3%

Table S5 - Quartiles and interquartile range for ensemble ranking

Individual networks

AGRIS regulatory network

The Arabidopsis Gene Regulatory Information Server (AGRIS) supplies a resource for gene regulatory studies for Arabidopsis thaliana. A component of AGRIS, the Arabidopsis thaliana regulatory network database (AtRegNet) consists of transcription factors (TFs) and their direct target genes only (Yilmaz et al., 2011). The AtRegNet database was converted into a network, keeping for each TF its direct target genes.

MaMut genetic modification design network

This network was extracted from the “genetic modification dataset” of CORNET, a publicly available database on gene associations in plants (De Bodt et al., 2012). This network contains information on differentially expressed genes when comparing wild type plants to transgenic plants. Links in this network represent genes that are either up- or downregulated when knocking out one or more transcription factors. These differentially expressed genes are assumed to be the target (either direct or indirect) of the transcription factor that was knocked out.

Protein-protein interaction (PPI) network

The PPI network was extracted from CORNET, and includes predicted as well as experimentally identified protein-protein interactions in Arabidopsis from different sources. Some of these interactions were derived from the original AraNet network (De Bodt et al., 2012).

GeneMANIA network

The GeneMANIA network represents a combination of different publicly available data sets, collected from a variety of databases. These include co-expression data, co-localization data, genetic interactions, physical interactions, shared protein domains and predicted interactions, all combined into a single network. A detailed

overview of all networks used by GeneMANIA can be found on the GeneMANIA website[1].

GENIE3 predicted regulatory network

In order to construct computationally predicted transcriptional regulatory networks, we used the GENIE3 algorithm (Huynh-Thu et al, 2009), which achieved the best performance on the DREAM5 network inference challenge (Marbach et al., 2012). GENIE3 was run on the "leaf" compendium from the CORNET database. The result of this analysis is a list of predicted transcription factor – target relations and an associated confidence score. Using a cutoff of 0.02 we only used the most confident associations to build the network.

Co-expression network using Pearson correlation coefficient (PCC)

Using the "leaf" compendium from the CORNET database we calculated a co-expression network by calculating the Pearson correlation coefficient between the expression patterns of all genes. As the resulting network is huge, we only keep the 5% most significant gene pairs, corresponding to the correlations that have at least an absolute value of about 0.8, thus keeping the most correlated or anti-correlated genes.

Text mining network

A network of gene-gene associations predicted using text mining was extracted from the Evex text mining resource (Van Landeghem et al., 2011). Text mining was built on top of PubMed abstracts and PubMed Central full text articles, covering over 40 million bio-molecular events among more than 76 million automatically extracted gene/protein name mentions. The text mining data further has been enriched with gene identifiers and gene families from Ensemble and HomoloGene, providing

[1] http://genemania.org/

homology-based event generalizations. In this work, we only used gene-gene associations that were annotated as belonging to Arabidopsis genes.

Network integration

Integrated network

Except for the PPI, MaMut and AGRIS networks, all other networks have weighted edges. To assemble all networks into an integrated one, we disregarded all weights and just kept all edges. Note that for the GENIE3 and PCC networks, the networks were already pre-filtered by using only the most confident edges. In terms of directedness, Evex, MaMut, AGRIS and GENIE3 are directed networks, while PCC, PPI and GeneMANIA are undirected networks. In order to integrate all networks, the integrated network thus constitutes an undirected network. In addition, self-loops, occurring only in the Evex and MaMut networks, have been removed. All computations related to network properties as well as integration were mostly done using two R packages: "*igraph*" (Csardi and Nepusz, 2006) and "*Matrix*" (Bates and Maechler, 2012).

Topological features

Two major classes of features were extracted from the network. General topological properties only capture the topology of genes in the network, and similarity measures compute how much a gene is similar to a set of pre-defined genes. The similarity can be measured either using topology information or using GO terms.

General topological properties

The following general topological properties were used for the prioritization approaches:

- <u>Degree</u>
  The most elementary characteristic of a gene is its degree (or connectivity), *k*, which tells us how many links the gene has to other genes (Junker and Schreiber, 2008)

- Betweenness Centrality
  Suppose that, in order for gene *i* to contact gene *j*, gene *k* must be used as an intermediate station. Then gene *k* is such that it has a certain "responsibility" to gene *i* and *j*. If we count all of the minimum (short) paths which pass through gene *k*, then we have a measure of "stress" which gene *k* must undergo during the activity of the network (Freeman, 1977). We can then calculate the total number of paths passing through the gene *k*, defined as

  $$C_B(p_k) = \sum_i^n \sum_j^n b_{ij}(p_k) \qquad i < j$$

  where *n* is the number of genes in the graph and $b_{ij}(p_k) = \frac{g_{ij}(p_k)}{g_{ij}}$ is the number of shortest paths linking p_i and p_j, and $g_{ij}(p_k)$ is the number of shortest paths linking p_i and p_j that contain p_k.
- Kleinberg's hub and authority scores
  The Kleinberg's hub and authority scores are based on the principle eigenvector of the adjacency matrix A of the network. The authority score is defined as the principle eigenvector of A^TA and the hub score is based on the principle eigenvector of AA^T (Kleinberg, 1998).
- Closeness
  The closeness of a gene is the inverse of the average length of the shortest paths to/from all the other genes in the graph (Freeman, 1978).

Topology-based similarity measures

- Number of shared genes with the known genes
  This number represents the number of neighbouring genes that a gene has in common with the known genes. This idea is based on the fact that, when two genes share many neighbours with each other, they are likely to be involved in the same biological process.
- Number of direct connections to the list of known genes
  This represents the number of edges of each gene directly connected to class GR genes
- Jaccard Similarity Index
  The Jaccard index, also known as the Jaccard similarity coefficient, is a statistic used for comparing the similarity and diversity of sample sets. The Jaccard coefficient measures similarity between sample sets and is defined as the size of the intersection divided by the size of the union of the sample sets:
  $J(A, B) = \frac{|A \cap B|}{|A \cup B|}$
  In a network, the Jaccard similarity coefficient of two genes is the number of common neighbors divided by the number of genes that are neighbors of at least one of the two genes being considered.
- Dice similarity index
  Related to the Jaccard index, the Dice index expresses the degree to which two different species are associated. The similarity of two sample sets A and B

is twice the intersection divided by the sum of the elements in two sets:

$$s = \frac{2|A \cap B|}{|A| + |B|}$$

In a network, the Dice index of two genes is twice the number of common neighbors divided by the sum of the degrees of the genes (Dice, 1945).

- Inverted weight score

  The inverse log-weighted similarity index was proposed to mine information on the internet in order to extract social networks, and is defined as:

$$\text{Similarity}\ (A, B) = \sum_{\text{Shared items}} \frac{1}{\log[\text{frequency}(\text{shared item})]}$$

  In a network, the inverse log-weighted similarity index of two genes is the number of their common neighbors, weighted by the inverse logarithm of their degrees. It is based on the assumption that two genes should be considered more similar if they share a low-degree common neighbor, since high-degree common neighbors are more likely to appear even by pure chance. Isolated genes will have zero similarity to any other vertex. Self-similarities are not calculated (Adamic and Adar, 2003).

- Shortest path

  The shortest distance between a gene and all class A genes can be considered a similarity index which can show some relatedness between a gene and the genes in class A.

GO-based similarity measures

For the approach based on GO term overlap, we define three term overlap similarity measures: term overlap between neighbours of a query gene and the seed genes, term overlap between the query gene itself and the seed genes, and finally the combination of two.

The magnitude of the term overlap was measured by the Jaccard similarity coefficient. For the approach based on GO enrichment, we followed the approach proposed in (Rahmani et.al, 2012). In this method, we count relevant GO terms for the set of seed genes S, and select the top ten statistically most overrepresented terms (Table S3). Next, for these terms a two-way table is constructed using the frequency of the terms in the seed genes as well as the query gene and its neighbors.

The reason for including neighbors is the fact that GO annotations of proteins can often be predicted well from the GO annotations of their neighbors (Schwikowski et al., 2000, Rahmani et al., 2011). The p-value of a Fisher exact test, comparing the frequency of terms in the two groups is then used as a similarity measure. The higher the p-value, the more similar the representation of GO terms between the two groups will be. The same procedure was then also applied for the top five and the first GO terms.

Model-based approaches

Model types

Naïve Bayes

A Naïve Bayes classifier uses Bayes' theorem, additionaly making the assumption of independence between features, given the class variable, to reduce the computational cost. Although it may seem that the independence assumption ignores relationships between features, most of the time Naïve Bayes performs well. We computed the conditional posterior probabilities for each class and consider the probability for class S to rank the test genes. The method was implemented by the R-package "*e1071*" (Dimitriadou et al., 2011).

Linear Discriminant Analysis (LDA)

LDA is a statistical classifier which tries to find the best linear combination of features in order to separate two classes of events. In the package "*MASS*" (Venables and Ripley, 2002), we used the lda function to fit the LDA. The prior probabilities of class membership are the class proportions for the training set. The posterior probability that a gene belongs to class S is the product of the prior probability and the multivariate normal density.

Support Vector Machines (SVM)

SVM, a machine learning algorithm, is a non-probabilistic binary classifier. The basic idea in SVM is to find a maximal margin separation between the two classes. SVM is able to manage non-linear classification problems by means of the so-called kernel trick.

The package "*e1071*" (Dimitriadou et al., 2011) was used to fit the SVM model. The kernel used is the default one, radial basis, the degree=3, gamma parameter defined 1/(data dimension), tolerance of termination criterion (default: 0.001), epsilon in the insensitive-loss function (default: 0.1), the shrinking option was used as well. To produce probabilistic outputs, we used the probability option in this package.

Lasso and elastic-net regularized generalized linear models (Glmnet)

The elastic net is a regularized regression model that uses two penalization criteria, L1 and L2. It combines these two criteria which come from lasso and ridge methods. We used the "*glmnet*" (Friedman et al., 2010) R package to fit a regularized version of logistic regression. This approach fits a generalized linear model by using penalized maximum likelihood. The regularization path is computed for the lasso or elasticnet penalty at a grid of values for the regularization parameter lambda. The lambda parameter is fixed to 0.9.

Random Forests (RF)

Random Forests are an ensemble of many single decision trees. The method uses a voting systems to determine the class of objects based on the output of singles trees. We used the random forests implementation of the "*randomForest*" R package (Liaw and Wiener, 2002). The number of trees used was the default value; 500. The number of variables randomly sampled at each split is the square root of the number of features. Sampling was done with replacement, and the sample size was chosen equal to the training set size. We used the vote ratio as a probability that a gene is a

member of class S. The vote ratio for class S is the number of trees that predicted class *S* for a given gene, divided by the total number of trees in the random forests model.

Generalized Boosted Regression Models (GBM)

GBM uses boosting, an iterative process to add new functions in order to reduce the misclassification rate. We used the "*gbm*" R package (Ridgeway, 2012), which implements extensions to Freund and Schapire's AdaBoost algorithm and J. Friedman's gradient boosting machine. For classification problems, the distribution was defined for "adaboost" (the AdaBoost exponential loss for 0-1 outcomes). All other parameters were fixed to their default values.

Ensemble methods

To explore to which extent the results of the model based classifiers could be improved we also tested different combinations of classifiers, a technique commonly referred to as ensemble models. Two main approaches to create ensembles were considered. First, by averaging the prediction probability of each classifie and second, by aggregating the ranks of the genes resulting from each classifier. We defined ten ranking ensembles as well as nine probability prediction ensembles. An overview of all combinations of methods that we tested can be found in Table S4. Overall, no major improvements were noted by combining methods, and the best combination was only able to slightly improve the median ranking from 589 to 520 (RF+Glmnet, Table S5). The result of the best first quartile (rank 127) was not improved upon by any combination of methods.

Network importance

To assess the importance of each subnetwork for the prioritization we explored the impact of leaving out each network, and compared the prioritization results without the network to the original approach using all networks integrated. Results are presented for the model-based methods (Figure S7) and the best ensemble method (Figure S8). The first box in each plot shows the ranking when we used the complete integrated network. The other boxes show the ranking when we removed the corresponding network from the integrated one. A decrease in the ranking compared to the first box (Total) shows that the corresponding network has a negative impact on the final ranking since removing the subnetwork allows the GR genes to be better ranked.

References

Yilmaz, A., Mejia-Guerra, M.K., Kurz, K., Liang, X., Welch, L., Grotewold, E. (2011) AGRIS: the Arabidopsis Gene Regulatory Information Server, an update. Nucleic Acids Res 2011, **39**(Database issue): D1118–D1122.

De Bodt, S., Hollunder, J., Nelissen, H., Meulemeester, N., Inzé, D. (2012) CORNET 2.0: integrating plant coexpression, protein-protein interactions, regulatory interactions, gene associations and functional annotations. New Phytol 2012, 195(3):707-20

Huynh-Thu, V.A., Irrthum, A., Wehenkel, L., Geurts, P. (2010) Inferring Regulatory Networks from Expression Data Using Tree-Based Methods. PLoS ONE 2010, 5(9): e12776.

Marbach, D., Costello, J.C., Küffner, R., Vega, N.M., Prill, R.J., Camacho, D.M., Allison, K.R., The DREAM5 Consortium, Kellis, M., Collins, J.J., Stolovitzky, G. (2012) Wisdom of crowds for robust gene network inference. Nature Methods 9:796–804.

Van Landeghem, S., Ginter, F., Van de Peer, Y., Salakoski, T. (2011) EVEX: A PubMed-Scale Resource for Homology-Based Generalization of Text Mining Predictions. In Proceedings of the 2011 Workshop on Biomedical Natural Language Processing: 23-24 June 2011; Portland, Oregon, USA. 28-37.

Csardi, G., Nepusz, T. (2006) The igraph software package for complex network research. InterJournal 2006, Complex Systems: 1695. http://igraph.sf.net

Bates, D., Maechler, M. (2012) Matrix: Sparse and Dense Matrix Classes and Methods. R package version 1.0-6 2012. http://CRAN.R-project.org/package=Matrix

Junker, B.H., Schreiber, F. (2008) Analysis of Biological Networks (Wiley Series in Bioinformatics). New York: Wiley; 2008.

Freeman, L.C. (1977) A set of Measures of Centrality Based on Betweenness.

Sociometry 1977, 40(1):35-41.

Kleinberg, J. (1998) Authoritative sources in a hyperlinked environment. In Proceedings of the Annual ACM-SIAM Symposium on Discrete Algorithms: 1998; ACM, New York. 668 – 667.

Freeman, L.C. (1978) Centrality in Social Networks Conceptual Clarification. Social Networks 1978/79, 1:215-239.

Dice, L. R. (1945) Measures of the Amount of Ecologic Association Between Species. Ecology 1945, 26(3):297-302.

Adamic, L.A., Adar, E. (2003) Friends and neighbors on the Web. Social Networks 2003, 25:211-230.

Rahmani, H., Blockeel, H., Bender, A. (2012) Predicting Genes Involved in Human Cancer Using Network Contextual Information. Journal of Integrative Bioinformatics 2012, 9(1):210.

Schwikowski, B., Uetz, P., Fields, S. (2000) A network of protein-protein interactions in yeast. Nat Biotechnol 2000, 18(12):1257–1261.

Rahmani, H., Blockeel, H., Bender, A. (2011) Collaboration-based function prediction in protein-protein interaction networks. In IDA'11 Proceedings of the 10th international conference on Advances in intelligent data analysis X: 2011; Berlin, Heidelberg. Edited by J. Gama, E. Bradley and J. Hollmen: Springer-Verlag; 2011: 318-327.

Dimitriadou, E., Hornik, K., Leisch, F., Meyer, D., Weingessel, A. (2011) e1071: Misc Functions of the Department of Statistics (e1071). TU Wien. R package version 1.6 2011. http://CRAN.R-project.org/package=e1071

Venables, W. N., Ripley, B.D. (2002) Modern Applied Statistics with S. New York: Springer; 2002.

Friedman, J., Hastie, T., Tibshirani, R. (2010) Regularization Paths for Generalized Linear Models via Coordinate Descent**.** Journal of Statistical Software 2010, 33(1): 1-22.

Liaw, A., Wiener, M. (2002) Classification and Regression by randomForest. R News 2002, 2(3): 18-22.

Ridgeway, G. (2012) gbm: Generalized Boosted Regression Models. R package version 1.6-3.2 2012. http://CRAN.R-project.org/package=gbm
